# Post-percutaneous nephrolithotomy pseudo aneurysm formation treated by coil embolization; A study of seven cases

**DOI:** 10.34172/jcvtr.32905

**Published:** 2024-03-13

**Authors:** Niki Tadayon, Meisam Refaei, Sina Zarrintan, Saleh Shahsavari, Doras Najari, Mohsen Sheikhzadeh

**Affiliations:** ^1^Clinical Research Developmental Unit of Shohada Tajrish Hospital, Shahid Beheshti University of Medical Sciences, Tehran, Iran; ^2^Department of General & Vascular Surgery, Shohadaye Tajrish Hospital, Shahid Beheshti University of Medical Sciences, Tehran, Iran; ^3^Division of Vascular & Endovascular Surgery, University of California, San Diego,California,USA; ^4^Department of Surgery, Shohada Tajrish Hospital,Shahid Beheshti University of Medical Sciences, Tehran, Iran; ^5^School of Medicine, Shahid Beheshti University of Medical Sciences, Tehran, Iran

**Keywords:** Coil embolization, Percutaneous nephrolithotomy, Renal artery

## Abstract

Renal artery pseudoaneurysm is a rare complication of percutaneous nephrolithotomy (PCNL) with symptoms of flank pain and hematuria. Endovascular coil embolization has been proposed as a safe management option. We report Seven male patients, aged 36 to 65 years, with post-PCNL pseudoaneurysms presenting as gross hematuria. They all underwent CT angiography prior to endovascular intervention. The access was from common femoral artery in 6 cases and from left brachial artery in one case. Selective angiography of affected renal artery and branches were performed by suitable catheter. Coil embolization was performed by MicroNester and MReye coils (Cook, Inc.). Size of coils was selected based on angiography results. Completion angiography revealed embolized pseudoaneurysm in all cases. Gross and microscopic hematuria disappeared in all patients in the following days. Endovascular angioembolization with coil is an effective technique for managing post-PCNL pseudoaneurysms in renal artery and its branches.

## Introduction

 The management of renal stone disease over the years has evolved from open pyelolithotomy to laparoscopic pyelolithotomy to percutaneous nephrolithotomy (PCNL) to retrograde intra-renal surgery.^1^

 PCNL is now recommended as first-line therapy for renal stones > 2 cm.^2^ While PCNL is considered a safe and effective procedure, it is still associated with the very serious complication of renal hemorrhage. Bleeding after PCNL occurs as a result of either traumatized renal parenchyma or injury to the perinephric vessels.^3^

 Age, sex, BMI, the presence of comorbidities, such as hypertension, diabetes mellitus, serum creatinine level, history of prior renal surgery, the degree of hydronephrosis, stone burden, number of punctures, inexperienced surgeon, large nephroscope and increase of intraoperative puncture time are factors that increase the probability of injury.^4^

 Renal vessel damage with subsequent development of arteriovenous fistulas or pseudoaneurysms is a well-known source of bleeding after PCNL.^5^ Arteriovenous fistulas and pseudoaneurysms of the renal arteries are formed by a high-pressure leak from a lacerated artery, which is transmitted through the tract into a lower-resistance system, such as a vein or a connective tissue space.^5^ When a pseudoaneurysm becomes larger and blood leaks into the renal collecting system, macroscopic hematuria can occur.

 This hemorrhage may occur immediately after the procedure or in the form of “delayed bleeding” which may occur after a few days.^6^ The majority of patients settle with conservative management. However, a small proportion of patients requires intervention in the form of angioembolization.^6^

 The primary diagnosis of these patients who have delayed and prolonged hematuria is by CT angiography, which can see the pseudoaneurysm or AVF.^7^

 The standard indications for DSA are recurrent or continuous bleeding with a drop in corrected hemoglobin of > 30 g/L or hemodynamic instability.^8^ The timing of intervention is based on clinical judgment and lack of response to resuscitation. Instability of vital signs and continuous bleeding are the basis of this decision.

 Previous studies have found that embolization rates vary from 0.6% to 2.6%, and transfusion rates vary from 1% to 11% after PCNL.^9^

 Super selective transcatheter arterial embolization (TAE) has been recommended as a safe and effective method for severe hemorrhage after PCNL.^10^

## Description of The Cases

###  Study design

 Data from 852 patients who underwent PCNL at our institution between 2016 and 2022 were retrospectively reviewed. Our patients included 580 males with the mean age of 49.7 and 272 females with the mean age of 51.1 years old.

**Figure 1:**
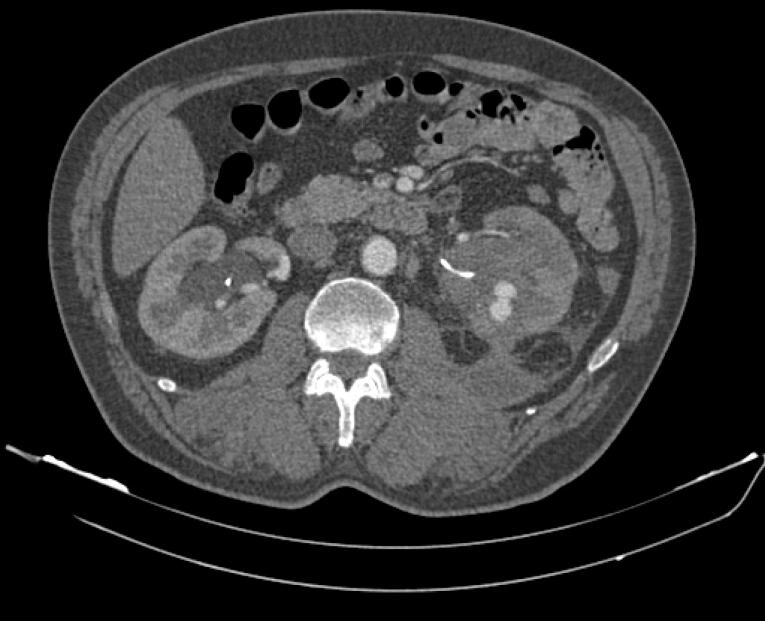
CTA showing a renal artery branch pseudoaneurysm

 Of these, 7 of them had significant postoperative gross hematuria that did not respond to supportive cares. Us being a referral vascular unit, these were referred to us between 4 and 14 days from the index procedure by the urology department. Detailed history and physical examination findings were noted from the case records. Regarding past medical history, two patients had diabetes and two patients had hypertension. None of the patients had a history of coagulation disorder and none of them were taking anticoagulant drugs. These patients were initially stabilized with intravenous fluids and blood transfusion. Preoperatively, hemogram, serum creatinine, serum electrolytes, coagulation profile, blood sugar levels, urine culture and CT angiography were performed in all patients. Evidence of pseudoaneurysm was seen in these 7 patients. (Figure 1)

###  Percutaneous Nephrolithotomy Technique

 All PCNL procedures were performed in the prone position with fluoroscopic assistance. All surgeries were performed with a rigid nephroscope. (Wolf 22 Fr with 20° lens). Stones were fragmented with a pneumatic lithotrite and by the end of the procedure, a double pigtail ureteric stent was placed in cases of large stone burden, injury to the pelvicalyceal system, bleeding, and residual calculi. A postoperative hemogram was performed in all patients immediately after the procedure and repeated after 24 hours. Additional checks were performed according to the patient’s situation and history. On the first postoperative day, a KUB was taken to confirm the double j’s placement.

 Of all these patients, seven had delayed postoperative hematuria, ranging from four days to eight weeks. These patients were initially stabilized with intravenous fluids and blood transfusion. Then routine assessments and labs were performed. Following informed consent, patients who had normal serum creatinine and severe immediate or delayed bleeding underwent an abdominal CT angiography before further procedures and evidence of pseudoaneurysm was seen in these patients.

###  Angioembolization Technique

**Figure 2:**
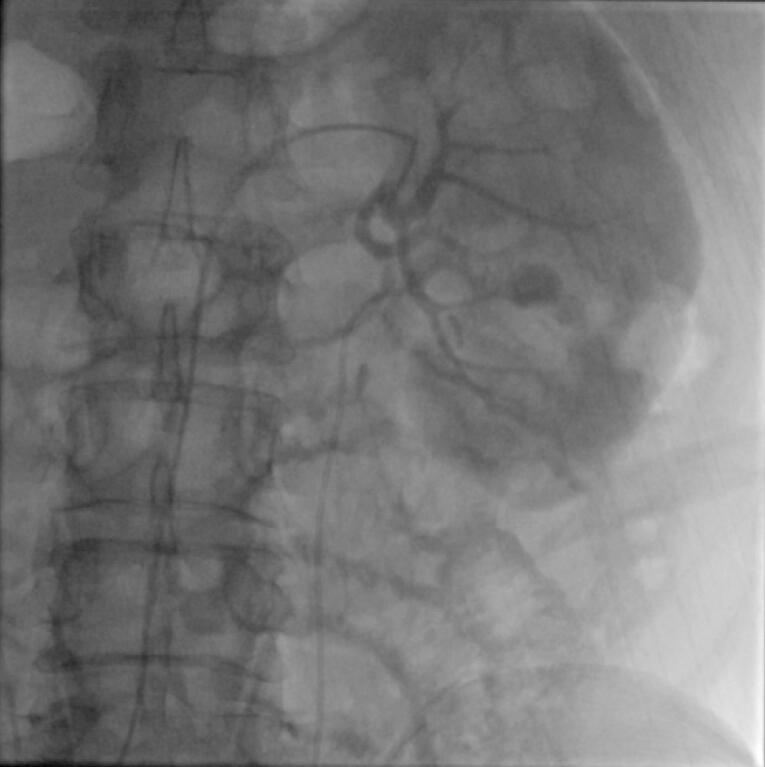
Selective angiography showing a renal artery branch pseudoaneurysm

**Figure 3:**
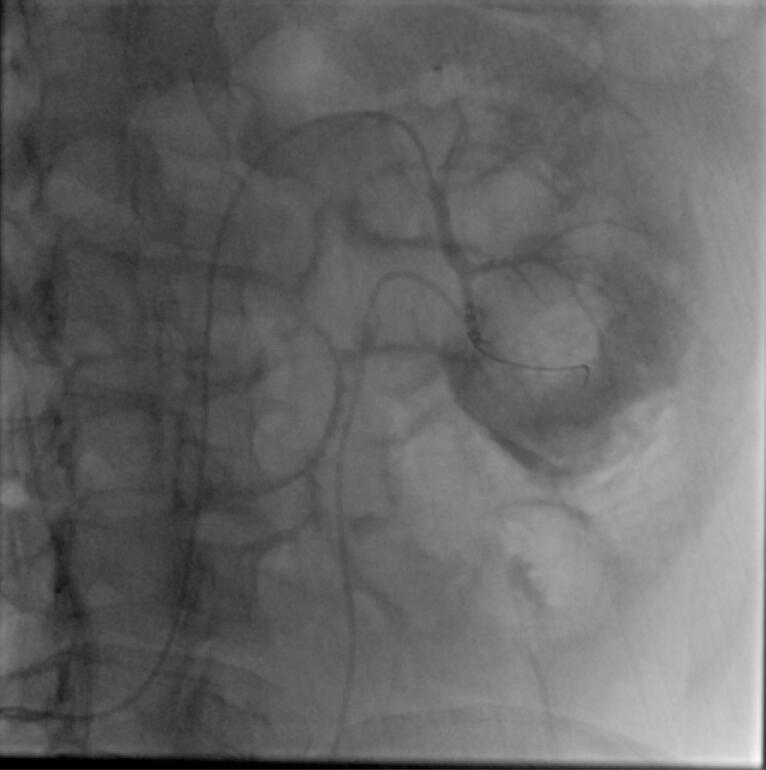
Angiogram documenting resolution of pseudoaneurysm after coiling

 The access was from common femoral artery in 6 cases and it was from left brachial artery in one case by using 6 French (Fr) femoral or Radial sheath respectively. All cases were performed under local anesthesia. Angiography of aorta and renal arteries was performed by a Pigtail 5Fr catheter followed by road mapping. Visualization of a saccular shape contrast along the artery translated to pseudoaneurysm. Figure 2 Selective angiography of affected renal artery and branches were performed by Cobra, Vertebral and MP catheters. Then microcatheter (2.7 Fr) and 0.018 or 0.014 guidewires were used to reach the bleeding artery and passed into the pseudoaneurysm. Coil embolization was performed by MicroNester and MReye coils (Cook, Inc.). Size of coils was selected based on angiography results considering the size and volume of the pseudoaneurysm. Table 1 tabulates the procedural characteristics of the patients. Completion angiography revealed embolized pseudoaneurysm in all cases. Blush of contrast material was not seen in any of the cases. Figure 3 Gross and microscopic hematuria disappeared in all patients in the following days, which we defined as clinical success. These patients were monitored closely, and complete blood counts and renal function tests were performed daily until stabilization.

**Table 1:** Characteristics of patients with intra-parenchymal renal artery pseudoaneurysms in the patients

**No.**	Interval*	**Access **	**Involved artery**	**Size**	**Coils**
1	2 weeks	Right FA	Left inferior pole	1 × 1 cm	5 mm & 8 mm
2	6 days	Right FA	Left superior pole	1 * 1 cm	5 mm & 8 mm
3	8 weeks	Left BA	Left inferior pole	1.5 × 1.5 cm	8 mm & 12 mm
4	10 days	Right FA	Left inferior pole	1 * 1 cm	2 × 5 mm
5	5 days	Right FA	Right superior pole	1.5 × 2 cm	2 × 5 mm & 8 mm
6	4 days	Left FA	Right inferior pole	1 * 1 cm	4 × 3 mm
7	5 days	Right FA	Right renal artery	1.5 × 2 cm	3 × 5 mm

*Internal between percutaneous nephrolithotomy (PCNL) and performance of coil embolization *BA* – Brachial Artery; *FA* – Femoral Artery

###  Analysis

 We analyzed pre-, intra-, and post-operative variables of the patients who required angioembolization. The preoperative factors included age, sex, BMI and presence of comorbidities such as diabetes mellitus, hypertension, and history of coagulation disorder or use of anticoagulant drugs. Other laboratory factors such as serum creatinine level, urine culture, degree of pelvicalyceal dilatation, cortical thickness and stone size, and burden were also assessed. Intraoperative factors included number of access tracts, involved artery, bleeding leading to hypotension and injury to the pelvicalyceal system. Postoperatively, we analyzed the fall in hemoglobin compared to the preoperative level, requirement of blood transfusion and/or inotropes for hemodynamic resuscitation, and the day of presentation for delayed bleeding. We also studied the timing of angioembolization post-surgery and the angiographic findings and outcomes of embolization.

 Stone size was defined as the greatest dimension for a single stone and summation of the greatest dimensions of all stones for multiple stones. The Guy’s stone score was used to assess stone burden.

 Data was analyzed in Microsoft Excel software (Microsoft Corporation, Washington, USA). Fisher’s exact test and Chi-square test were used in univariate analysis. Logistic regression analysis was used in multivariate analysis with a value of P < 0.05 considered statistically significant.

## Results

 Out of 852 patients who underwent PCNL, 512 experienced mild hematuria which resolved one or two days after the operation. The rest got better after conservative management. However, seven out of the 852 patients (0.82%) required angioembolization to control bleeding. They presented with tachycardia and hypotension and all required blood transfusion. These seven patients, were all male and between 36 and 65 years old.

 None of the patients had a history of coagulation disorder or were taking anticoagulant drugs. The patients were initially stabilized with intravenous fluids and blood transfusion. The lowest BMI was 23 (kg/m2) and the highest was 32(kg/m2). There was no difference in the type of stones. Three cases had multiple stones and three had single stones. One patient also had staghorn stone. Two patients had diabetes and two patients had hypertension.

## Discussion

 PCNL is a safe and effective operation for renal stones. Bleeding is the most common and significant complication of PCNL,^11^ with the reported incidence of bleeding requiring transfusion as high as 20%, with 7% as the average incidence.^9^

 Risk factors for severe hemorrhage post-PCNL requiring embolization included UTI, hypertension, diabetes mellitus, number of tracts, and stone type.^5^

 Arterial injuries usually appear as pseudoaneurysm or AVF. Pseudoaneurysm is a perfused hematoma contained by the adventitia and perivascular tissues that is in communication with the lumen of an adjacent artery and due to the higher pressure of the artery than the urinary collecting system, it leads to gross hematuria. Hematuria due to arterial injury usually does not improve. Therefore, we should always consider arterial injuries in patients with persistent, severe hematuria.

 Thus, Patients with prolonged and delayed hematuria, hematocrit drop and lack of response to supportive care should be imaged. CT angiography is the imaging method of choice for arterial injuries in patients with prolonged gross hematuria after PCNL.^12^ Contrast material or fistula can be seen in CTA. Pseudoaneurysms caused by PCNL usually develop in the terminal branches of the renal artery.

 Angiography and embolization have been established for severe, persistent, or intermittent hemorrhage post-PCNL that cannot be stopped by conservative treatment.^13,14^ Due to damage in the terminal branches, Angioembolization should be considered as a first-line management tool. The method is effective and can reduce mortality and morbidity, and usually kidney function is not damaged.

 In our series the technical and clinical success rate was 100%. The post-procedural courses were uneventful. In the follow-up period, rebleeding did not occur. Thus, endovascular coil embolization is a safe and effective technique in the management of post-PCNL pseudoaneurysms in renal artery and also in branches of renal arteries.

 The limitation of our study was the small number of patients. On the other hand, all patients needed Angioembolization. Patients without complications or with little and controlled hematuria were not referred to our department.

## Conclusion

 Hematuria following PCNL is one of the inevitable complications of this procedure. Performing the procedure by an experienced person as well as reducing the risk factors is effective in reducing injuries. However, it is vital to recognize arterial injuries that require intervention.

 Today, Angioembolization is the most appropriate and best treatment method for patients with persistent gross hematuria caused by PCNL who have not responded to supportive treatment.

## Competing Interests

 The authors have no conflict of interest.

## Ethical Approval

 Ethics code number: IR.SBMU.RETECH.REC.1401.094.

## Funding

 None.
